# Imaging mass cytometry of the immune microenvironment in alveolar echinococcosis

**DOI:** 10.3389/fcimb.2026.1759455

**Published:** 2026-05-08

**Authors:** Yanyan Ma, Yaogang Zhang, Tao Zhang, LingQing Zhang, Zhixin Wang, Zihan Yang, Xinjian Guo, Jingqi Han, Jing Hou, Meiyuan Tian, Dengliang Huang, Haijiu Wang, Haining Fan

**Affiliations:** 1 Research and Management Department, Qinghai University Affiliated Hospital, Xining, Qinghai, China; 2 Qinghai Provincial Key Laboratory of Hydatid Disease Research, Qinghai University Affiliated Hospital, Xining, Qinghai, China; 3 Qinghai Provincial Hydatid Disease Clinical Medical Research Center, Qinghai University Affiliated Hospital, Xining, Qinghai, China; 4 Central Laboratory, Qinghai University Affiliated Hospital, Xining, Qinghai, China; 5 Department of Pathology, Qinghai University Affiliated Hospital, Xining, Qinghai, China

**Keywords:** alveolar echinococcosis, echinococcus multilocularis, imaging mass cytometry, immune, immune microenvironment

## Abstract

**Background:**

Alveolar echinococcosis (AE), caused by Echinococcus multilocularis, is a severe parasitic disease exhibiting tumor-like growth and profound immune evasion. The spatial organization and immunosuppressive mechanisms within the human AE lesion microenvironment remain poorly characterized.

**Methods:**

High-dimensional imaging mass cytometry (IMC) was performed on liver tissue sections from six patients with histologically confirmed AE, comparing lesion-proximal (close liver tissue, CLT) and lesion-distal (distant liver tissue, DLT) regions. This approach enabled simultaneous profiling of immune cell infiltration, spatial heterogeneity, immune checkpoint expression, and microenvironmental architecture. Complementary immunofluorescence and immunohistochemistry were used to evaluate age-related differences in neutrophil subsets between pediatric and adult patients.

**Results:**

Sixteen immune cell types were resolved by IMC, revealing distinct spatial distributions: CD15- neutrophils and CD4+ effector memory T (Tem) cells accumulated in partially necrotic zones; M1/M2 macrophages and dendritic cells formed ring-like structures around cytotoxic T lymphocyte (CTL)-rich areas; CD4+ and CD8+ effector T cells exhibited dispersed circular patterns. M2 macrophages showed a negative correlation with T cells—suggesting T cell exhaustion—and a positive association with CD15+ neutrophils but not CD15-neutrophils. Immune checkpoint molecules were generally expressed at low levels in CLT, with focal PD-1/PD-L1 co-expression, broad CD47 distribution, rare NKG2A^+^ cells, and TIGIT enrichment in innate NK cells. Based on the TIMELASER classification system, the AE immune microenvironment was categorized into three subtypes: marrow-enriched immunosuppressive, stroma-enriched immunosuppressive, and immune-resident. Significantly higher densities of both CD15+ and CD15- neutrophils were observed in CLT of pediatric patients compared to adults.

**Conclusion:**

This study provides the first spatially resolved map of the immune landscape in human AE, revealing dynamic immunosuppressive niches shaped by myeloid–lymphoid crosstalk and checkpoint regulation. These findings offer new insights into early immune evasion mechanisms and potential targets for host-directed therapeutic strategies.

## 1 Introduction

Alveolar echinococcosis (AE) is a severe zoonotic parasitic disease caused by the larval stage of *Echinococcus multilocularis* (*E. multilocularis*), resulting in grave health repercussions among afflicted individuals. It is predominantly prevalent in the Northern Hemisphere, including Europe, North America, and Asia (Bresson-Hadni et al., 2021; Wen et al., 2019). Notably, the epidemiology of AE has recently exhibited multifaceted dis-semination and expansion trends, with a substantial rise in previously unmonitored countries (Kui et al., 2021; Wang et al., 2025). The larval stage of *E. multilocularis* involves invasion of the liver, causing hepatic alveolar echinococcosis (HAE). HAE has a long incubation period of 5–15 years (Kern, 2021), with latent and invasive growth. In the early stages, there are often no clinical symptoms; however, in the late stages, it is diagnosed due to the occurrence of various complications, which are more severe. The difficulty of surgery is known as the “Mount Everest” of hepatobiliary and pancreatic surgery (Autier et al., 2023; Brunetti et al., 2010). Over 18,000 new cases of AE are estimated to emerge globally each year, with the vast majority, 91%, taking place in China (Casulli et al., 2019; Fu et al., 2021; Torgerson et al., 2010). HAE has particularly gained prominence in the Qinghai–Tibet Plateau region of China. A survey conducted by the Chinese Center for Disease Control and Prevention via ultrasound screening revealed an alarming 10.95% positivity rate for HAE in some regions of the Qinghai–Tibet Plateau. Apart from gender, the highest positivity rate was found among individuals aged 10 to 14 years, with a range of approximately 1.47% to 1.7%. In contrast, the positive rate in the 20 to 79-year age group was lower, approximately 0.5% (Zheng et al., 2020). Initially, patients often experience an absence of symptoms, leading to delayed diagnosis owing to the emergence of various complications, for example, the presence of an enlarged liver, obstructive jaundice, and portal hypertension. If left untreated, 90% of cases may result in mortality within 10–15 years after diagnosis (Autier et al., 2023; Brunetti et al., 2010). AE not only poses significant challenges to public health and well-being but also imposes a substantial economic burden on families and society at large (Lundstrom-Stadelmann et al., 2025). Hence, conducting in-depth research elucidating the immune mechanisms of AE and exploring immunotherapeutic targets is of paramount importance.

Traditional proteomic techniques predominantly offer insights into the global protein expression of cells or tissues. In contrast, innovative imaging mass cytometry (IMC) technology enables the con-current acquisition of high-resolution cell images and multiparameter protein expression data from tissue samples, furnishing a wealth of spatial and molecular information. Researchers have additionally employed IMC technology in hepatocellular carcinoma (HCC) research, revealing diverse topological functional units within the HCC tumor microenvironment (TME) and constituting the largest pathology landscape repository for HCC. These advancements have spurred breakthroughs in the realm of HCC immunotherapy (Sheng et al., 2022). In the field of parasitic infection research, scholars have harnessed the potential of IMC, coupled with cytokine measurement, bulk RNA-seq transcriptomic analysis, and machine learning, to investigate the immunological characteristics and dynamic immune responses to malaria infections in both European and African populations. Through this, essential markers of acquired immune responses have been identified, facilitating analysis of cell function and thereby propelling the development of effective vaccines in malaria-endemic regions (de Jong et al., 2021). The immune response to AE is complex, and the specific mechanisms involved remain unclear (Zhang et al., 2003). The innate immune system, which acts as the body’s first line of defense against *E. multilocularis* infection, has, until recently, been the primary focus of investigations concerning individual cells such as macrophages and T cells. However, the immune response induced by AE is a dynamic, multicellular process. Introducing spatial omics techniques may provide us with a more intriguing perspective to elucidate its mechanisms.

In this study, we employed advanced spatial omics technology to comprehensively characterize the immune microenvironment in hepatic alveolar echinococcosis. Our research aimed to elucidate the complex cellular composition, spatial organization, and interactive networks within the immune landscape surrounding *E. multilocularis* lesions. By investigating immune cell infiltration patterns, inter-cellular relationships, and immune checkpoint expression profiles in the hepatic tissue microenvironment, we seek to uncover the immunological mechanisms underlying the parasite’s ability to establish persistent infection. These insights may ultimately contribute to the development of novel immuno-therapeutic strategies for this challenging parasitic disease.

## 2 Materials and methods

### 2.1 Patient and methods

A total of 21 patients with a pathological diagnosis of AE were included in this study. The age range of the children group was 0–18 years old, and that of the adult group was 19–69 years old. The minimum age of the final collected cases was 7 years old, and the maximum was 60 years old. All patients were recruited from the Qinghai University Affiliated Hospital. Written informed consent was obtained from all the adult patients or parents of the children. The study protocol was approved by the Ethics Committee, and samples required for subsequent experiments were obtained after surgical resection. For high-dimensional spatial profiling of AE lesions, pathological tissues from six patients (three adults and three children) were selected for imaging mass cytometry (IMC) (**Supplementary Table 1**). Each sample was divided into two parts: close liver tissue (CLT, less than 0.5 cm from the lesion) and distant liver tissue (DLT, more than 2.5 cm from the lesion). The tissues used for IMC were fixed in formalin, embedded in paraffin, and cut into 3 μm sections. To validate the IMC findings related to neutrophil subsets, immunohistochemistry (IHC) and immunofluorescence (IF) analyses were performed on samples from all 21 patients. Frozen tissue sections were prepared at a thickness of 3 μm for these validation experiments.

### 2.2 Imaging mass cytometry staining and acquisition

**Figure 1:**
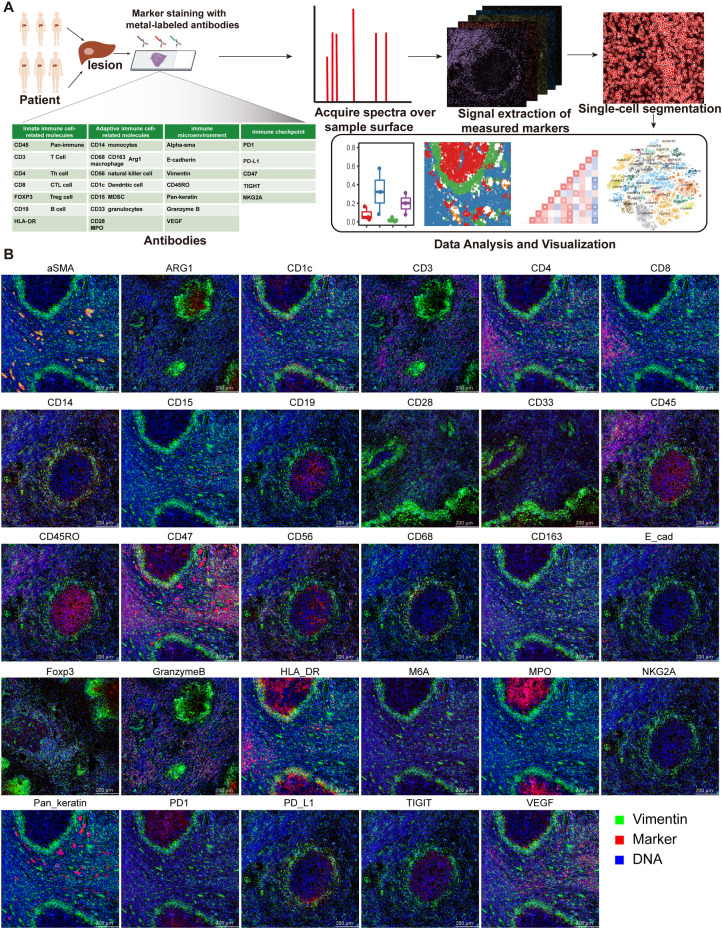
Workflow of imaging mass cytometry. **(A)** Imaging mass cytometry workflow: DLT and CLT regions were selected from the liver pathological sections of 3 adults and 3 children with AE and labeled with metal-tagged antibodies, and the spectral signals on the sample surface were obtained. The cells were segmented via bioinformatics technology for data analysis and visualization. **(B)** Distribution of individual marker protein expression; scale bar=200 μm.

**Figure 2:**
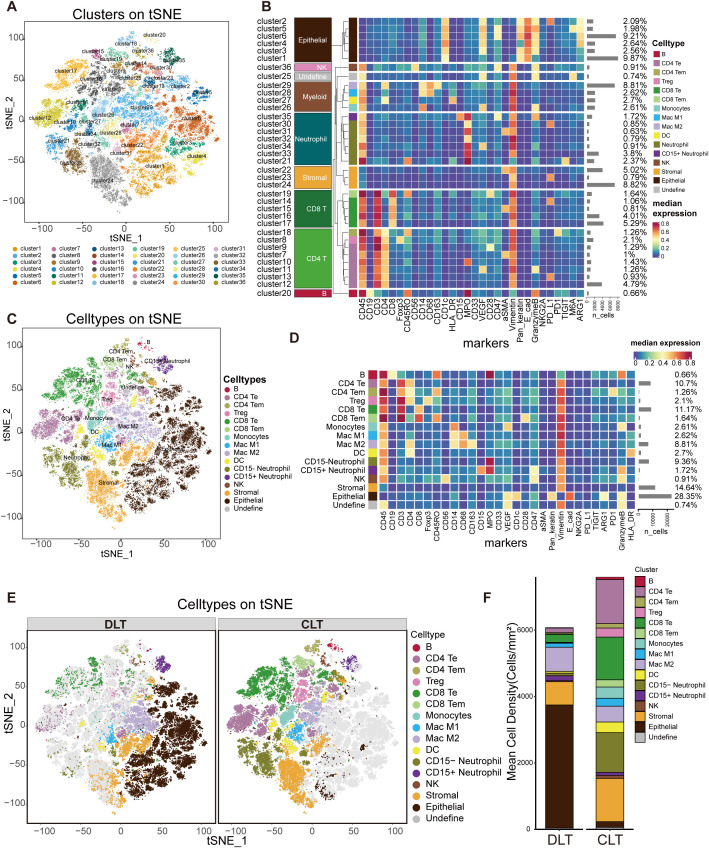
Data-driven derivation of cell phenotypes. **(A)** Cluster results obtained by tSNE dimension reduction analysis; **(B)** cluster heatmap and classification results of cells according to the expression of each cluster marker protein; **(C)** after cell classification was obtained, tSNE dimension reduction analysis was used to obtain the cell type clustering results.**(D)** Cell type clustering heatmap and the proportion of each cell; **(E)** tSNE dimensionality reduction analysis results of cells in the DLT and CLT regions (n=6); **(F)** Mean density(cells/mm^2^) of various types of cells in the DLT and CLT regions (n=6).

**Figure 3:**
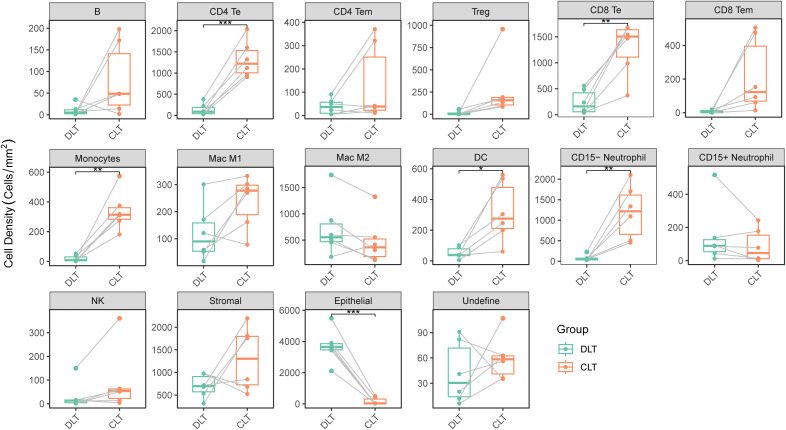
Spatial heterogeneity and quantification of cells in the immune microenvironment surrounding lesions in AE patients. Histograms of the cell densities comparison of each cell in the DLT and CLT regions (n=6) via Wilcoxon test. * *P* < 0.05; **P < 0.01; ***P < 0.001.

**Figure 4:**
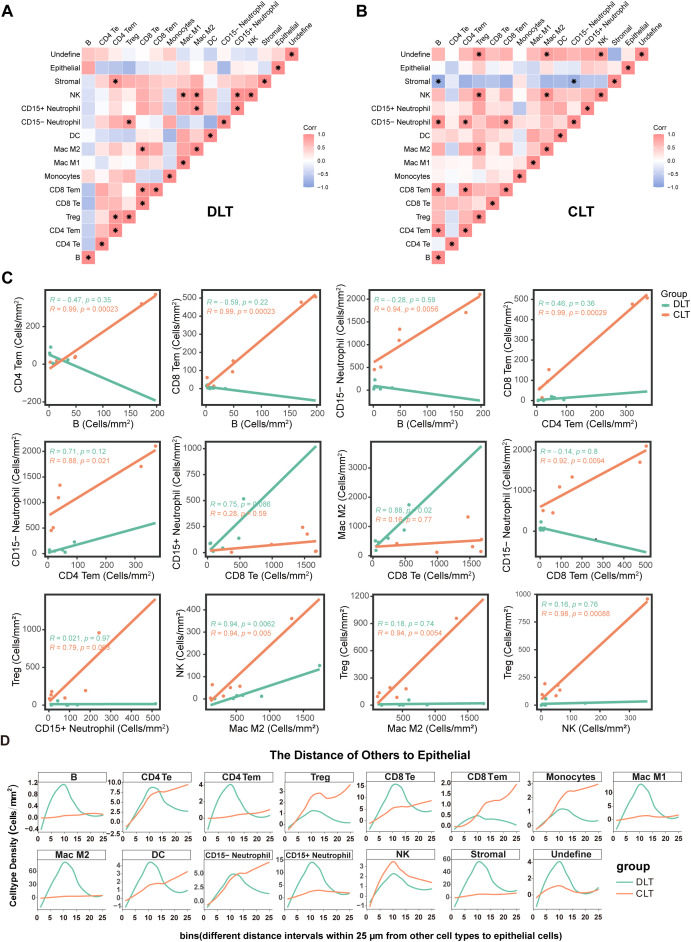
Spatial topological relationship of immune microenvironment cells around lesions associated with hepatic alveolar echinococcosis. **(A, B)** The density of cell type subsets in each sample in the DLT regions and the CLT regions was determined, and the Pearson correlation coefficient of each cell subset was calculated according to the density of different sample cell types. The symbols 1 (red) and -1 (blue) represent positive and negative correlations, respectively. The asterisk indicates that the difference is statistically significant, and the absolute value of the correlation coefficient is greater than 0.6. **(C)** Correlation analysis of different cell types in all regions revealed that the absolute value of the Pearson correlation coefficient exceeded 0.6. **(D)** The upper epithelial cells were employed as the reference point to calculate the distance between other cells and epithelial cells. Calculated the number of cells of a certain type within a 25μm range from the nearest epithelium, with each 1μm segment defined as an interval, to see the proportion of a certain type of cell within that interval out of the total number of that type of cell within the 25μm.

**Figure 5:**
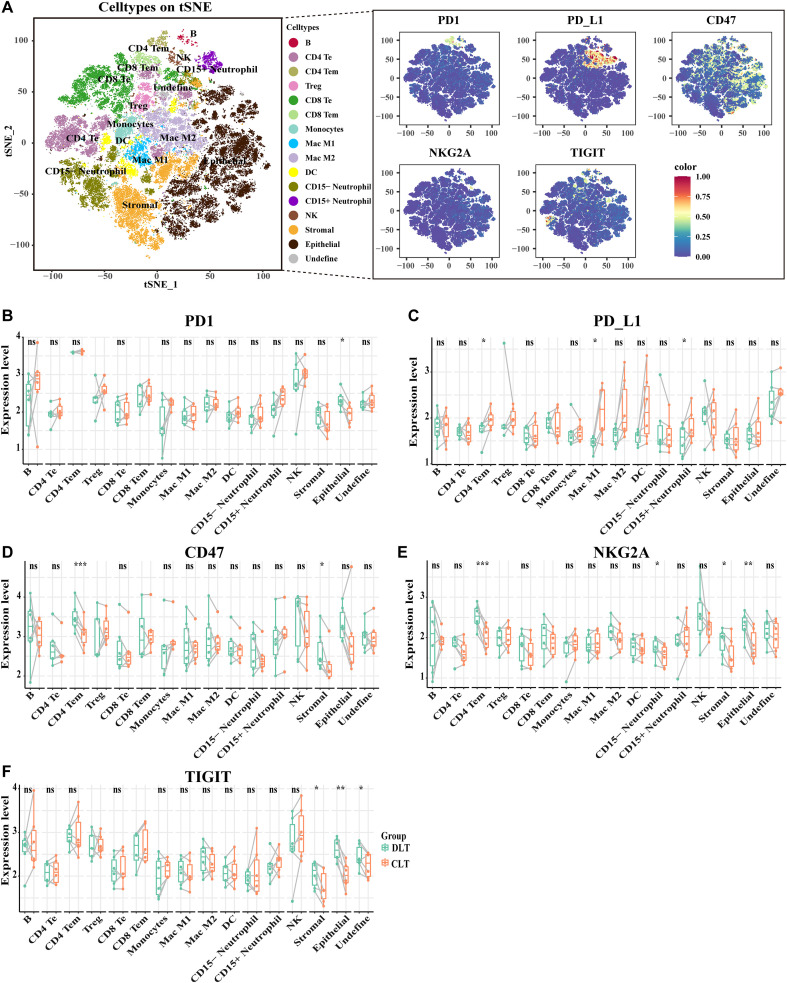
Spatial heterogeneity of immune checkpoint molecules in AE patients. **(A)** Feature plots showing the overall distribution of PD-1, PD-L1, CD47, NKG2A, and TIGIT signals on the cell tSNE map. Panel A is intended for qualitative visualization of signal distribution rather than quantitative comparison between cell types. **(B–F)** Boxplots showing the relative expression levels of PD-1 **(B)**, PD-L1 **(C)**, CD47 **(D)**, NKG2A **(E)**, and TIGIT **(F)** across annotated cell types in the DLT and CLT regions. These panels were used for quantitative comparison of checkpoint expressions between regions and cell populations. Paired sample t test, two-sided (n=6).

**Figure 6:**
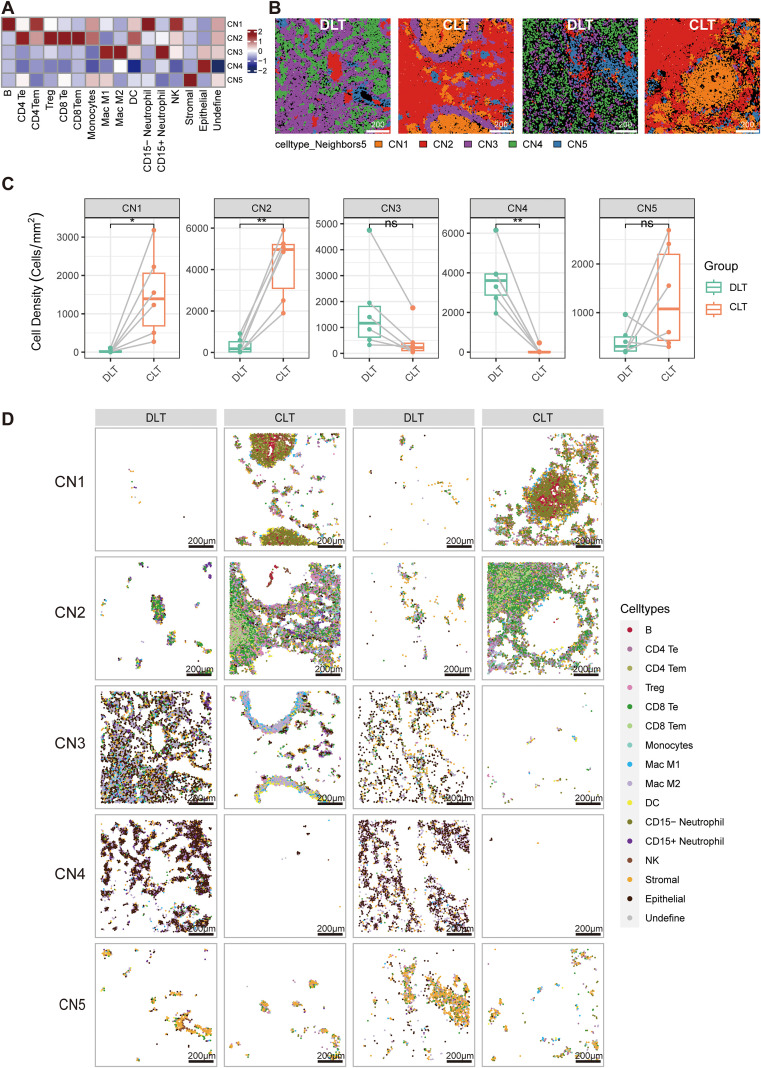
Immune microenvironment network of hepatic alveolar echinococcosis. **(A)**The heatmap of cellular neighborhoods obtained using the K-nearest neighbor (KNN) algorithm shows which types of cells were mainly composed in each CN. **(B)** The KNN algorithm was used to calculate the cell type components surrounding each cell, and the immune microenvironment was classified via hierarchical clustering (n=5), with nodes representing cell types and connecting lines representing the type of immune microenvironment in which they are located. **(C)** Cell density of the immune microenvironment types and intergroup comparisons were made via paired sample t tests (n=6). ns, not statistically significant; *P < 0.05; **P < 0.01. **(D)** Distribution of various types of cells in the CN1-CN5 region in the DLT and CLT regions in **Figure 6B**.

**Figure 7:**
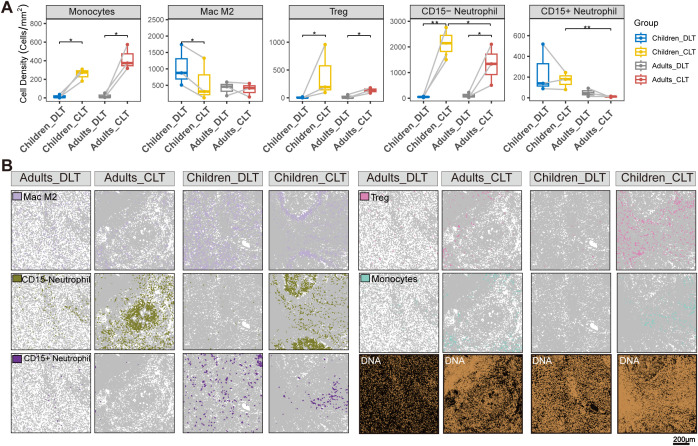
Cell distributions in the CLT and DLT regions between pediatric and adult patients. **(A)** Boxplots of cell density in the DLT and CLT regions of patients with AE (n=6); One-way ANOVA or paired samples t test, two-sided; ns, no statistical significance; * *P* < 0.05; *P < 0.01; ***P < 0.001. **(B)** Pseudocellar map of the cell distribution in the tissue imaging scan image in **(A)** grey=other cell types.

Imaging mass cytometry was performed to comprehensively characterize the spatial immune landscape of AE lesions, including immune cell composition, tissue localization, and immune checkpoint expression. This method corresponds to the main spatial analyses presented in **Figures 1**–**7**. IMC was performed using the Hyperion™ Imaging System (Fluidigm) according to the manufacturer’s standard protocol for FFPE sections (PN 400322 A3). This technology enables simultaneous detection of 30 protein markers using metal-tagged antibodies, providing spatially resolved single-cell data.

#### 2.2.1 Tissue preparation and staining

FFPE sections (3 μm) were first baked for 2 hours at 60 °C in a slide oven to remove paraffin residues. Dewaxing was performed in fresh xylene for 20 minutes in a fume hood, followed by hydration through descending grades of ethanol (100%, 95%, 80%, and 70%), 5 minutes each. Slides were then washed in Maxpar Water for 5 minutes with gentle agitation on an orbital shaker. For antigen retrieval, slides were incubated in preheated Antigen Retrieval Reagent (pH 9.0, 1× diluted from 10× stock) at 96 °C for 30 minutes using a heating block. Following antigen retrieval, slides were cooled to 70 °C (approximately 10 minutes), then washed sequentially in Maxpar Water (10 minutes) and Maxpar PBS (10 minutes) with gentle agitation. The tissue sections were blocked with 3% bovine serum albumin (BSA) in Maxpar PBS for 45 minutes at room temperature in a hydration chamber. A PAP pen was used to encircle the tissue area to retain reagents.

#### 2.2.2 Antibody panel

A custom-designed panel of 30 metal-conjugated antibodies (Maxpar^®^ metal-labeled antibodies, Fluidigm) was employed to characterize immune cell subsets, stromal components, and immune checkpoint molecules (**Supplementary Table 2**). The panel included markers for: (1) innate immunity (CD15, CD68, HLA-DR, etc.); (2) adaptive immunity (CD3, CD4, CD8, CD20, etc.); (3) immune checkpoints (PD-1, PD-L1, CD47, NKG2A, TIGIT); and (4) structural markers (E-cadherin, Vimentin, α-SMA). The antibody cocktail was prepared by diluting individual antibodies in Maxpar PBS containing 0.5% BSA according to the manufacturer’s recommended dilutions. The cocktail was centrifuged at 13,000 × g for 2 minutes, and the supernatant was carefully pipetted from the top to avoid aggregates. Slides were incubated with the antibody cocktail overnight at 4 °C in a hydration chamber.

#### 2.2.3 DNA staining and washing

After primary antibody incubation, slides were washed twice in 0.2% Triton X-100 in Maxpar PBS (8 minutes each) followed by two washes in Maxpar PBS (8 minutes each). Nuclear staining was performed using Cell-ID™ Intercalator-Ir (1:400 dilution in Maxpar PBS, 300–500 μL per section) for 30 minutes at room temperature. Slides were then washed in Maxpar Water for 5 minutes and air-dried for at least 20 minutes at room temperature.

#### 2.2.4 Image acquisition

Regions of interest (ROIs) were identified using corresponding hematoxylin-eosin (H&E) stained serial sections. Selected slides were loaded into the Hyperion Imaging System ablation chamber. The tissue was ablated with a high-energy ultraviolet laser (spot size ~1 mm²), and the ablated material was transferred to a time-of-flight mass spectrometer for metal isotope detection. For each patient, 6 ROIs were selected (3 from CLT and 3 from DLT regions), with consistent ROI areas across samples. Sections from the previous step were incubated for 2 hours at 60 °C and then dewaxed in xylene. The slides were hydrated in various concentrations of ethanol (100%, 95%, 80%, 70%) for 5 min each. Finally, antigen retrieval was performed at 96 °C with 1× antigen retrieval solution (pH 9.0) for 30 min. After cooling to 70 °C, the slides were blocked at room temperature with DPBS containing 3% BSA (pH 7.2-7.4) for 45 min. Then, the slides were incubated overnight with an antibody mixture (Maxpar^®^ Metal-labeled antibody) prepared with DPBS containing 0.5% BSA (pH 7.2-7.4) at 4 °C. The next day, the slides were washed with DPBS (pH 7.2-7.4), and the tissue was stained with an Ir intercalator (201192A 1:400) for 30 min at room temperature. The slides were washed and dried for the next step. Regions suitable for IMC were identified via hematoxylin–eosin (HE) staining.

### 2.3 Data processing

Raw IMC data were processed using a standardized image-analysis pipeline to extract single-cell information and identify immune cell populations in AE tissues. This section corresponds to the analytical workflow underlying **Figures 1**–**7**. Data were acquired with CyTOF software (Fluidigm) coupled to a Hyperion™ Imaging System. ROIs were defined according to the ablation coordinates, and the protein expression of each ROI was visualized using MCD™ Viewer (v1.0.560.2, Fluidigm). Probability maps were generated after training via Ilastik (v1.4.0) (Berg et al., 2019). The segmentation of the nuclei and cell boundaries was performed with CellProfiler (v4.1.2) (Carpenter et al., 2006) via the watershed module. The protein expression of a single cell was defined as a cell feature, calculated using histoCAT (v1.7.6) (Schapiro et al., 2017) on GitHub. The cell type definition was based on protein expression. For single-cell analysis, the batch effect was removed via Harmony (v0.1.0) (Korsunsky et al., 2019). Rphenograph (v0.99.1) (https://github.com/JinmiaoChenLab/Rphenograph) was used for cell clustering and is also available on GitHub. Heatmaps were generated via ComplexHeatmap (v2.10.0) (https://github.com/jokergoo/ComplexHeatmap) to show the median Z score (0--1) of the marker expression of the cells in each cluster. To reduce the dimension, we use visual T-random neighbor embedding (t-SNE) to determine the phenotypic diversity of the cell populations. Spatial analyses were further conducted to characterize the organization of the AE immune microenvironment. Cytomapper (v1.6.0) was used for processing and visualizing multiple multichannel images and cell boundaries. ImcRtools (v1.1.7) was used for spatial analysis and neighborhood analysis. Cell populations were annotated according to marker expression patterns and mapped back to the original tissue sections for downstream analyses of cell density, spatial association, immune checkpoint distribution, and immune microenvironment architecture.

### 2.4 Immunohistochemistry and immunofluorescence validation

**Figure 8:**
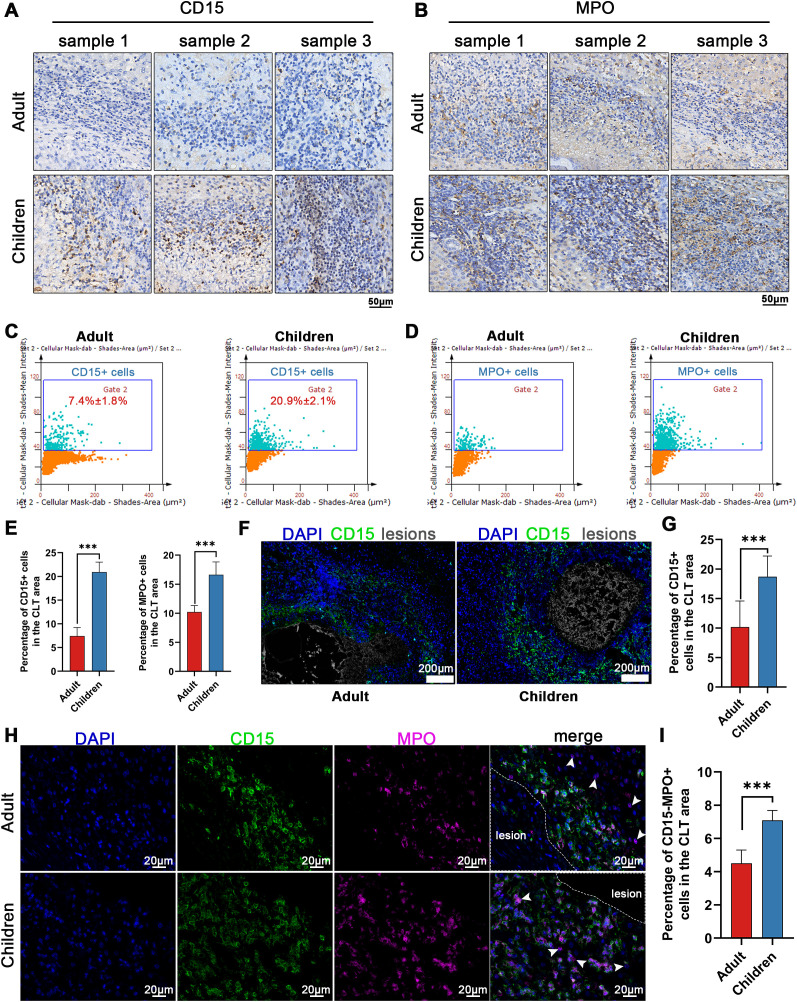
Distribution of Neutrophils in Hepatic Lesions of Pediatric and Adult Patients with A. **(A, B)** Immunohistochemistry results showing CD15, MPO positive neutrophils in the CLT region of hepatic lesions from pediatric (n=10) and adult (n=11) patients with AE. **(C, D)** Percentages of CD15, MPO positive neutrophils in the CLT region identified via Strata Quest software (three regions per patient). **(E)** Bar graph illustrates the percentages of CD15, MPO positive neutrophils in the CLT region. **(F, G)** Immunofluorescence results of CD15+ neutrophils in the CLT region of hepatic lesions in pediatric and adult patients with AE. **(H, I)** Immunofluorescence results of CD15-MPO+ neutrophils in the CLT region of hepatic lesions of pediatric (n=10) and adult (n=11) patients with AE, the white arrow indicates CD15-MPO+ cells. Independent Samples t-Test, two-sided; ***P < 0.001.

IHC and IF were performed to validate the IMC-based observation that neutrophil subsets were differentially enriched in lesion-adjacent tissue, particularly in pediatric patients. This section mainly corresponds to the validation results shown in **Figure 8**.

#### 2.4.1 Immunofluorescence

Frozen sections were dried at room temperature, fixed for 10 min in cold acetone, and placed in EDTA antigen retrieval buffer for 10–15 min. The samples were washed three times for 5 min each with PBS (pH 7.4). The sections were incubated with 3% hydrogen peroxide solution at room temperature in the dark and then blocked with 2% goat serum in PBS for 30 min. CD15 protein was detected with an anti-CD15 antibody (Abcam, ab16285, 1:200) and stained with a secondary antibody (Goat Anti-Mouse IgM H&L, Abcam, ab9167, 1:10000). MPO protein was detected with an anti-MPO antibody (Abcam, ab45977, 5 µg/ml) and stained with a secondary antibody (Goat Anti-Rabbit IgG H&L, Abcam, ab205718, 1:20000). The nuclei were stained with DAPI Staining Solution (Abcam, ab228549), antifade mounting medium was added, and the slides were sealed. Images were acquired using a TG TissueFAXS fluorescence microscope.For IF-based interpretation, CD15+ cells were used to identify CD15+ neutrophils, whereas CD15−MPO+ cells were used to represent the CD15-negative neutrophil-related population identified in the IMC analysis.

#### 2.4.2 Immunohistochemistry

To further confirm the tissue distribution of CD15 and MPO by chromogenic staining, immunohistochemistry was performed on corresponding tissue sections. The slides were dewaxed, and endogenous peroxidase blockers were added to the tissue. Subsequently, the tissue was incubated at room temperature for 10 min and washed with double-distilled water. For microwave-based antigen retrieval, the slides were placed in EDTA antigen retrieval solution (BOSTER, AR0023), heated for 8 min at high power in a microwave oven, allowed to cool naturally for 8 min, reheated for 8 min, and then cooled to room temperature.

The sections were incubated with 5% BSA blocking solution for 30 min at 37 °C. The primary antibodies (CD15 [MC-480], Abcam ab16285, 1:50; MPO, Abcam ab45977, 5 µg/ml) were incubated with the samples overnight at 4 °C. After removal of the primary antibody, the slides were rewarmed at 37 °C for 30 min and washed three times with PBS. Secondary antibody incubation was then performed using PolyHRP-conjugated anti-mouse (BOSTER, SV0001) or anti-rabbit (BOSTER, SV0002) IgG at 37 °C for 30 min, followed by three washes with PBS. A working solution of DAB chromogen was added, and the samples were observed under a microscope to determine the reaction time. The sections were counterstained with hematoxylin for 1 min at room temperature, returned to blue in alkaline solution, mounted with neutral gum, and scanned using the TissueGnostics HistoQuest system. For quantitative validation, three representative regions from the CLT area were analyzed per patient using StrataQuest software, and the percentages of CD15+ cells and CD15−MPO+ cells were compared between pediatric and adult patients.

### 2.5 Statistical analysis

All statistical analyses were performed using R software (version 4.1.0). Prior to parametric testing, data normality was assessed using the Shapiro-Wilk test and homogeneity of variances was evaluated using Levene’s test. For normally distributed data with equal variances, paired t-tests were used for within-subject comparisons (e.g., DLT vs. CLT regions from the same patient), while independent samples t-tests were employed for between-group comparisons (e.g., adult vs. pediatric patients). For data that violated parametric assumptions, non-parametric alternatives (Wilcoxon signed-rank test for paired data) were utilized.

When comparing more than two groups, one-way analysis of variance (ANOVA) was performed for normally distributed data, followed by Tukey’s honestly significant difference (HSD) *post-hoc* test for multiple comparisons. For spatial correlation analyses between cell types, Pearson correlation coefficients were calculated for normally distributed variables. Correlation coefficients with absolute values >0.6 and adjusted *P*-values <0.05 were considered significant. Statistical significance was de-fined as *P* < 0.05 (two-tailed). The R packages used included stats, lme4, ggplot2, pheatmap, and multcomp for specialized analyses.

## 3 Results

### 3.1 Spatially resolved phenotyping of hepatic alveolar echinococcosis ecosystems via IMC

To explore the cellular composition of HAE while preserving its spatial context, paraffin-embedded sample sections from 6 patients with AE were chosen. Within each paraffin section, a total of 6 regions consisting of three repeats of DLT and CLT regions were selected. An IMC panel was ingeniously devised to investigate HAE. This involved the use of IMC and a set of 30 protein antibodies that were conjugated with isotopically pure dilute metal reporter genes (see **Supplementary Table 2**). These selected antibodies aimed to elucidate the involvement of innate immunity, adaptive immunity, and stromal and immune checkpoint proteins (**Figure 1A**), with a specific emphasis on their role in disease progression. Random forest classifier (Ilastik) and cell image analysis software (CellProfiler) were used to isolate individual cells. A total of 82112 single cells from 348 images [more than 4500 cells for each group (**Supplementary Figure 1**)] were acquired after segmentation of the cell boundaries and quality control. The median expression of each marker protein in each sample was further analyzed. The expression and distribution of each marker protein are shown in **Figure 1B**.

### 3.2 Data-driven derivation of cell phenotypes

Based on the density of each marker, we used a clustering algorithm to perform cell clustering with a phenograph (k=45) and identified 36 clusters (**Figure 2A**). We subsequently defined the cell types of these clusters since protein expression while each cluster was backfilled onto the original section for spatial localization analysis (**Supplementary Table 3**). As a result, the cells were roughly classified into 9 distinct types (**Figure 2B**): B cells, CD4 T cells, CD8 T cells, neutrophils, myeloid cells, NK cells, epithelial cells, stromal cells, and undefined cells (CD45+ and VEGF+ cells). We further divided CD4 T cells into CD4 effector T cells (Te), CD4 memory T cells (Tem), Treg cells, and CD8 T cells into CD8 Te cells and CD8 Tem cells. Similarly, myeloid cells differentiate into M1 macrophages (MAC M1), M2 macrophages (MAC M2), dendritic cells (DCs), CD15- neutrophils, CD15+neutrophils and NK cells. A total of 16 cell categories were obtained (including B cells, CD4 Te, CD4 Tem, Treg, CD8 Te, CD8 Tem, myeloid cells, MAC M1, MAC M2, DC, CD15- neutrophils, CD15+neutrophils, NK cells, epithelial cells, stromal cells, and undefined cells) (**Figures 2B, C**). Because the cells were backfilled into the sections, the epithelial cells were predominantly hepatocytes. Within the DLT and CLT regions, neutrophils (9.36%) and MAC M2 cells (8.81%) constituted a relatively high proportion of innate immune cells, whereas NK cells (0.91%) were the least abundant. In contrast, CD4 Te (10.7%) and CD8 Te (11.17%) accounted for a relatively greater proportion of adaptive immune cells, with B cells (0.66%) being the least numerous (**Figures 2C, D**). There was a marked distinction in the density of DLT and CLT cell types, with the DLT region being primarily epithelial, and some immune cells, mainly MAC M2, CD8 Te, and CD4 Te cells. The CLT region was primarily composed of immune cells, with a relatively high percentage of CD8 Te cells, CD4 Te cells, and CD15- neutrophils, followed by MAC M2, MAC M1, DC, and Treg cells (**Figures 2E, F**).

### 3.3 Spatial heterogeneity and quantification of immune microenvironment cells around hepatic alveolar hydatid disease lesions

After the cells were classified according to their location in the original section, A comparison of the densities of cells in the DLT and CLT regions revealed that the densities of CD4 Te cells, CD8 Te cells, CD8 Tem cells, monocytes, DCs, and CD15-neutrophils were significantly greater in the CLT region (*P <* 0.05). Conversely, the densities of epithelial cells were significantly lower in the CLT region (*P* < 0.05), whereas the densities of B cells, CD4 Tem cells, Tregs, MAC cells, and NK cells did not significantly differ between the DLT and CLT regions (*P* > 0.05) (**Figure 3**).

### 3.4 Spatial topological relationships of immune microenvironment cells surrounding lesions associated with hepatic alveolar echinococcosis

To understand the spatial distribution patterns of cell subtypes in HAE, we calculated the classification of the 20 nearest neighbors for each cell to determine the probability of occurrence for each cell subtype in the sample. The findings are presented as Pearson correlation coefficients, with red indicating positive correlations, blue indicating negative correlations, and darker shading indicating a greater number of regions of interest (ROIs) with significant interactions (**Figures 4A, B**). The results revealed that the interaction between B cells and CD4 Tem, CD8 Tem, and CD15- neutrophils in the CLT region was more robust than that in the DLT region (**Figures 4B, C**). The interaction between CD4 Tem cells and CD8 Tem cells, CD15-neutrophils within the CLT region was significantly enhanced (**Figures 4B, C**). In the case of macrophages, we found potential interactions between Mac M2 and CD8 Te cells in the DLT region and between Mac M2 and Treg in the CLT region. Additionally, we found a positive correlation between Treg cells and NK cells in the CLT region (**Figures 4B, C**). These findings indicate that macrophages and Treg cells, NK cells may contribute to the immune tolerance of AE. Another interesting finding is that CD15-neutrophils have a high positive interaction with B cells, CD4 Tem, and CD8 Tem in the CLT region (**Figures 4B, C**).

To gain further understanding of immune cell infiltration and comprehend the spatial heterogeneity of immune cells, we calculated the distance between other cells and epithelial cells (primarily hepatocytes) The total number of cells of a given cell type within 1 μm from the slice was counted. The region surrounding the lesion was analyzed at 25 μm. Many cells were concentrated at a depth of 5-15 μm within the DLT regions, for example, B cells, CD4 Tem, CD8 Te, Mac M1, Mac M2, DC, NK, CD15+ neutrophils and stromal. In the CLT region, the number of Treg cells, CD8 Tem, CD4 Te, monocytes, and CD15- neutrophils gradually increases within the range of 0-25μm (**Figure 4D**). However, the results above may be influenced by the different numbers of epithelial cells in DLT and CLT.

### 3.5 Spatial heterogeneity of immune checkpoints in AE

The immune checkpoints serve as crucial nodes in regulating the intensity of immune responses and are essential for maintaining immune system homeostasis and normal functioning. AE displays numerous similarities with tumors, an understanding of the distribution and expression of immune checkpoints within immune cells near AE lesions can provide new insights into the potential of immunotherapy for treating this condition.

To investigate checkpoint heterogeneity in the AE microenvironment, we analyzed the distribution and relative expression of PD-1, PD-L1, CD47, NKG2A, and TIGIT across the 16 annotated cell types. Importantly, **Figure 5A** shows the overall distribution of checkpoint signals on the tSNE map, whereas **Figures 5B–F** show the quantitative comparison of relative expression levels across cell types and between DLT and CLT regions.PD-1 signal was primarily observed in lymphoid populations on the tSNE map, with relatively prominent expression in CD4 Tem cells and NK cells. Quantitative analysis further showed that PD-1 expression did not differ significantly between DLT and CLT in most immune cell populations, whereas epithelial cells showed reduced PD-1 expression in the CLT region (**Figure 5B**). PD-L1 showed a heterogeneous distribution pattern on the tSNE map and was detectable in both immune and non-immune cell populations. In the quantitative analysis, PD-L1 was present across multiple cell types, with relative enrichment in several myeloid and lymphoid populations. In addition, CLT-associated increases were observed in selected cell populations, including CD4 Tem cells, M1 macrophages, and CD15+ neutrophils (**Figure 5C**).CD47 was broadly distributed across the tSNE map, consistent with its relatively ubiquitous expression pattern. Quantitative comparison showed that CD47 expression was reduced in CD4 Tem cells and stromal cells in the CLT region relative to the DLT region, while differences in most other cell types were limited (**Figure 5D**). NKG2A-positive signals were relatively restricted on the tSNE map. Quantitative analysis showed that NKG2A expression was most evident in NK cells, with additional but weaker expression in several other populations, including stromal and epithelial cells. Compared with the DLT region, NKG2A expression decreased in CD15− neutrophils, stromal cells, and epithelial cells in the CLT region (**Figure 5E**). TIGIT was mainly detected in lymphoid populations, particularly CD4 Tem, Treg, CD8 Tem, and NK cells. Quantitative comparison further indicated that TIGIT expression decreased in stromal cells, epithelial cells, and CD45+VEGF+ undefined cells in the CLT region (**Figure 5F**). Overall, these results indicate that immune checkpoint molecules in AE exhibit marker-specific and cell type-dependent heterogeneity, while most CLT-versus-DLT differences are selective rather than global.

### 3.6 Immune microenvironment network of hepatic alveolar echinococcosis

The classification of the immune microenvironment in AE remains uncertain, and we have referred to the TIMELASER system for the classification of the immune microenvironment in liver cancer. Considering the classifications, we employ the K-nearest neighbor (KNN) algorithm to determine the composition of the cell types in the vicinity of each cell. We subsequently utilized hierarchical clustering to categorize the immune microenvironment into five distinct cellular neighborhoods (CNs), designated CN1--CN5.(**Figures 6A, B**) Each immune microenvironment exhibited a considerable degree of differentiation (**Figure 6B**).CN1 and CN2 are primarily located in the CLT area, CN3 and CN5 are present in both CLT and DLT areas, and CN4 is mainly found in the DLT area(**Figure 6C**).CN1 is centrally situated within uniquified and necrotic lesions, primarily consisting of B cells, CD15-neutrophils,NK and few CD4 Tem cells, similar to the above immune residence phenotype. CN3 tight collar lesions are distributed in a ring and are predominantly composed of myeloid cells such as MAC M1, MAC M2, and CD15+neutrophils. This distribution is like that observed in the immune-suppressive myeloid phenotype, with a notable absence of adaptive immune cells, particularly T cells, which appear to be depleted. CN2 is distributed in a ring-like pattern alongside CN3, with a majority of CD4 Te, CD8 Te and Treg cells. This distribution is like that observed in the immune activation phenotype. CN4 is primarily localized in the DLT region, encompassing epithelial cells (predominantly hepatocytes), low-expressing DC and NK cells. This distribution is analogous to the immune exclusion phenotype in HCC, which is characterized by a deficiency of immune cells in this region. CN5 is composed primarily of stromal cells which are recognizable features of the immunosuppressive stromal phenotype. However, it also presents unique features, with a small number of MAC M2 cells dispersed among the stromal cells. It may play a role in the formation of an immunosuppressive microenvironment. (**Figures 6A, B, D**).

In essence, the TIMELASER typing system provides a framework for classifying the immune microenvironment of AE. The immune microenvironment of AE is exceedingly intricate and consists of five primary types: immune activation, immune-suppressive myeloid, immune-suppressive stromal, immune exclusion, and immune residence phenotypes.

### 3.7 Differences in neutrophil distribution within the immune microenvironment between pediatric and adult patients

Differences in immune function between pediatric and adult patients have been reported; however, in the context of AE, multiple factors—including infection duration, cumulative exposure, and disease stage—may influence immune responses. The adaptive immune system provides defense against pathogenic threats, which are often presented by antigen-presenting cells (APCs), while also establishing long-lasting protection through the formation of memory cells. Various APC subsets, such as dendritic cells, monocytes, macrophages, and granulocytes, exhibit reduced functionality at a young age. Altered APC functionality may consequently impact adaptive immune responses. Hence, we further analyzed the disparities in cell distribution between the DLT and CLT regions in pediatric and adult patients. Compared with those in adults, CD15+ neutrophils and Treg cells exhibited greater cluster formation in the CLT region of children (**Figures 7A, B**).In both adult and pediatric groups, the distribution of CD15- neutrophils in the CLT area is higher than in the DLT area (*P* < 0.05), and in the CLT area, the cell density of CD15- neutrophils in the pediatric group is higher than in the adult group(*P* < 0.05)(**Figures 7A, B**).

We found significant differences in the distribution of neutrophils within the AE lesions between adults and children, so we validated these findings by selecting pathological sections from 11 adult patients and 10 *pediatric* patients (**Supplementary Table 4**). The first intriguing finding is the discrepancy in the distribution of CD15+ neutrophils within the CLT region between adults and children. Thus, we employed immunohistochemistry and immunofluorescence to *analyze* the difference in the cell density of CD15+ neutrophils in the CLT region between adults and children via the CD15 protein. The results revealed a greater presence of CD15+ neutrophils in *pediatric* patients than in adult patients (**Figures 8A, C, E–G**). Using Strata Quest software, we subsequently determined the percentage of CD15+ neutrophils occupying the CLT region, which was significantly greater in *pediatric* patients than in adult patients (*P < *0.05) (**Figure 8E**). Finally, we employed immunofluorescence to further *analyze* the distribution of CD15+ neutrophils in the CLT region. The results also revealed a substantial accumulation of neutrophils around lesions in *pediatric* patients, surpassing the levels observed in adult patients (**Figure 8G**).

Another noteworthy finding is the aggregation of CD15-neutrophils within partially necrotic lesions, with higher levels observed in the CLT region than in the DLT region. To confirm this, we used CD15-MPO+ cells to represent CD15-neutrophils through Immunofluorescence. The distribution of CD15 and MPO positive cells in the CLT area was also *analyze*d using immunohistoc*hemic*al techniques. The results demonstrated a significant increase in CD15 and MPO positive cells in *pediatric* patients compared with adult patients(*P < *0.05) (**Figures 8A-F**). Furthermore, through the utilization of Strata Quest software, we identified a greater proportion of CD15-MPO+ cells occupying the CLT region in *pediatric* patients than in adult patients (*P < *0.05) (**Figures 8H, I**). These findings indicate that both CD15+ neutrophils and CD15-neutrophils are more abundant in the CLT regions of *pediatric* patients than in those of adult patients, suggesting a potential role for neutrophils in AE progression. Additionally, these findings highlight differences in the immune microenvironment between *pediatric* and adult patients; however, such differences may be influenced by multiple factors, including disease stage, infection duration, and host-specific variables.

## 4 Discussion

IMC technology uses metal-tagged antibodies to label tissue section samples, offering better insight into the phenotype and interactions of proteins and corresponding cells within the tissue microenvironment. In this study, we conducted an IMC analysis of immune infiltration, intercellular relationships in the immune microenvironment, and immune checkpoints in liver pathological samples from patients with hepatic AE for the first time. Compared with conventional flow cytometry and immunohistochemistry, IMC allows for simultaneous detection of more than 30 protein markers on both cell surfaces and intracellularly in a single scan, thus facilitating high-resolution single-cell analysis of the immune microenvironment in tissues.

Previous investigation revealed a ring-like distribution of DC cells, M1 macrophages, and M2 macrophages near the lesion (Wang et al., 2020). Anther studies also revealed that CD68+ macrophages clustered around lesions in liver samples and that the numbers of s100A9+ proinflammatory (M1 phenotype) and CD163+ anti-inflammatory (M2 phenotype) macrophages were significantly greater in the CLT than in the DLT, with M2 macrophages being the dominant macrophage group. In the early stage of infection, the M1 phenotype is dominant, whereas in the chronic stage of infection, the M2 phenotype of anti-inflammatory macrophages is dominant (Wang et al., 2020). DCs and macrophages phagocytose antigen particles released from the *Echinococcus* laminated layer (Diaz et al., 2023), which are presented to B cells and T cells to activate humoral or cellular immunity (An et al., 2024). We found that, adjacent to DC cells, M1 macrophages and M2 macrophages are CD4 T cells and CD8 T cells with a ring-like but dispersed distribution pattern, which is consistent with the sequential activation of innate immunity preceding adaptive immunity activation. This study revealed that the distribution of CD4+Te and CD8+Te cells was greater in the CLT region than in the DLT region. T cells are an important part of the immune microenvironment and are responsible for recognizing and eliminating pathogens or malignant cells. These functions are mediated by CD4 T cells, which are the main ‘regulators’ of the immune system, and CD8 T cells, which are the main ‘enforcers’ of the immune response (Soto-Heredero et al., 2021). However, some studies have found that continuous antigen stimulation can lead to T cells losing their effector functions and ultimately entering a state of dysfunction known as “T-cell exhaustion.” (Ou et al., 2025; Zhang et al., 2024). Hao Wen et al (Zhang et al. (2020)). found that *E. multilocularis* can induce liver T-cell exhaustion through the inhibitory receptor TIGIT and that blocking this immune checkpoint can reverse T-cell dysfunction, suggesting that immunotherapy targeting T-cell exhaustion may also be a method for treating AE.

Neutrophils, as the forefront of defines in the innate immune system, protect the host through phagocytosis, generation of reactive oxygen species (ROS), and release of neutrophil extracellular traps (NETs) to eliminate pathogens (Du et al., 2024; Knackstedt et al., 2019). Previous studies have shown an increase in CD15+ neutrophils following exposure to Toxoplasma gondii (T. gondii) tachyzoites or cysts (Bergersen et al., 2023). T. gondii tachyzoites can inhibit neutrophil apoptosis, thereby prolonging the lifespan of human neutrophils (Lima et al., 2021). In patients infected with Plasmodium falciparum, the number of circulating neutrophils increases, whereas the number of lymphocytes decreases (Aitken et al., 2018). Neutrophil activation is closely associated with the severity of human malaria (Kho et al., 2019; Prah et al., 2020). Similarly, we observed an abundance of CD15+ neutrophils in the CLT region of children with HAE. Compared with adults, children exhibit a greater distribution of NK cells and Treg cells in the CLT region, which may be associated with early-stage disease in children. Further analysis revealed a positive correlation between CD15+ neutrophils and M2 macrophages, which inhibited Th1 immune responses and promoted disease progression. Another interesting finding is the presence of numerous CD15- neutrophils and B cells in partially liquefied necrotic lesions. However, this inhibitory effect may be limited, or neutrophils and B cells recruited to the lesion may be antagonized or deactivated in the immunosuppressive microenvironment (Takeda et al., 2019; Xue et al., 2022). CD15- neutrophils are positively correlated with M1 macrophages. Scholars utilized the monoclonal antibody (mAb) Em2G11, which targets the Em2 antigen, to analyse paraffin-embedded specimens from AE or CE patients and discovered small particles of *E. multilocularis* (SPEMs) enveloped by numerous lymphocytes that were smaller than 1 µm and distant from the main lesion (Barth et al., 2012). These SPEMs containing CD15- neutrophils and B cells indicate that CD15- neutrophils and B cells play a role in the early immune response.

In addition, we found that neutrophils were more abundant in the CLT regions of pediatric patients than in those of adult patients, which may suggest a potential role for neutrophils in AE progression. Parasites, as well as their eggs, secretions, and other metabolic products, can serve as persistent antigens that continuously stimulate the host immune system, leading to sustained infiltration of immune cells such as neutrophils and prolonged release of inflammatory mediators, including cytokines and chemokines, thereby promoting chronic inflammation. Increasing evidence indicates that persistent inflammation is a key component of many diseases. Aberrant neutrophil recruitment and activation can not only cause direct tissue damage but also amplify the initial inflammatory response and contribute to the progression from acute to chronic inflammation (Filep, 2022; Gordon, 2016). Chronic inflammation is not only a hallmark of many parasitic diseases but also a central mechanism underlying the development of more severe complications, including cancer (Sorci and Faivre, 2009).

In addition, neutrophils can directly trap and kill parasites through the release of neutrophil extracellular traps (NETs), thereby limiting parasite dissemination and providing time for the development of subsequent immune responses. However, the role of NETs is double-edged: while they contribute to host defense, they may also participate in pathological tissue damage. For example, in Toxoplasma gondii infection, NETs can kill approximately 25% of parasites (Branzk and Papayannopoulos, 2013). In Leishmania infection, NETs restrict parasite motility and reduce parasite survival, but they may also exacerbate tissue injury and inflammation (Guimaraes-Costa et al., 2012). In malaria, NETs promote intravascular parasite aggregation and endothelial cell activation, thereby aggravating inflammatory pathology (Knackstedt et al., 2019). Higher neutrophil density in pediatric patients may be associated with enhanced inflammatory responses; however, whether this finding reflects greater disease activity or a distinct age-dependent immune response remains unclear.

Immune checkpoint expressions in the AE microenvironment showed a heterogeneous rather than uniformly elevated pattern. PD-1/PD-L1 displayed selective distribution patterns, CD47 was broadly distributed, NKG2A was relatively restricted, and TIGIT was mainly detected in lymphoid populations, especially NK- and T-cell-associated subsets. Importantly, the differences between DLT and CLT were not global across all cell types but were limited to selected populations. This suggests that checkpoint regulation in AE is spatially and cell type dependent, rather than representing a uniform immunosuppressive state throughout the lesion-adjacent tissue. Such heterogeneity is consistent with the complex immune architecture of AE and may reflect localized immune adaptation at the host–parasite interface. Hao Wen et al., utilizing single-cell RNA sequencing of peripheral blood, CLT regions, and DLT regions from four patients with cystic echinococcosis (CE), reported significantly increased expression of genes associated with immune suppression, particularly the immune checkpoint genes NKG2A/HLA-E, in the surrounding lesion tissue (Yasen et al., 2021). Research has indicated that during schistosomiasis infection in mice, the expression of NKG2A in both NK cells (Li et al., 2015) and NKT cells (Cha et al., 2017) is significantly reduced, suggesting the activation of NK cells. However, a study has reported downregulation of NK cells and upregulation of NKG2A expression on NK cells during *E. multilocularis* infection (Abulizi et al., 2019). In contrast, our study observed a lower expression of NKG2A around lesions in patients with AE, which may be attributed to the excessive noise resulting from sample size or heterogeneity. Compared with these previous reports, our IMC data suggest that NKG2A expression in AE is present but spatially heterogeneous, with quantitative enrichment most evident in NK cells and more limited expression in other populations. The discrepancy between studies may be related to differences in disease type, tissue compartment, analytical platform, and sample size. Taken together, these findings indicate that immune checkpoint regulation in AE is nuanced and context dependent. Rather than showing uniform checkpoint activation, the AE lesion microenvironment appears to contain localized checkpoint-defined niches, which may contribute to immune evasion and should be considered when evaluating potential host-directed immunotherapeutic strategies.

By applying imaging mass cytometry (IMC) to characterize immune cell infiltration, intercellular interactions, immune checkpoint expression, and the immune microenvironment of HAE at single-cell resolution, we provide new insights into the spatial heterogeneity of immune cells within lesion tissues and identify potential targets for diagnosis and immunotherapy. However, several limitations should be acknowledged. First, the sample size for IMC analysis was relatively small (n = 6), and the included patients may represent different disease stages and treatment backgrounds, which could introduce bias and limit the generalizability of our findings. Therefore, validation in larger, well-characterized cohorts is warranted. Second, the observed differences between paediatric and adult patients may be influenced by confounding factors—such as disease stage, infection duration, and prior treatment history—rather than age alone and should be interpreted with caution. Third, as this study is based on single time-point tissue sampling, it does not allow for causal inference or assessment of dynamic changes in the immune microenvironment during disease progression. Finally, the intrinsic technical limitations of IMC may introduce some degree of measurement deviation.

Taken together, further studies with larger cohorts, well-controlled clinical variables, and complementary methodologies, particularly longitudinal and functional analyses—are needed to validate and extend our findings.

## Data Availability

The original contributions presented in the study are included in the article/**Supplementary Material**. Further inquiries can be directed to the corresponding author.
