# Supplementary figures and images for: Imaging mass cytometry of the immune microenvironment in alveolar echinococcosis
Source: Front Cell Infect Microbiol. 2026 May 8;16:1759455. doi: 10.3389/fcimb.2026.1759455 (PMC13194580; doi:10.3389/fcimb.2026.1759455)

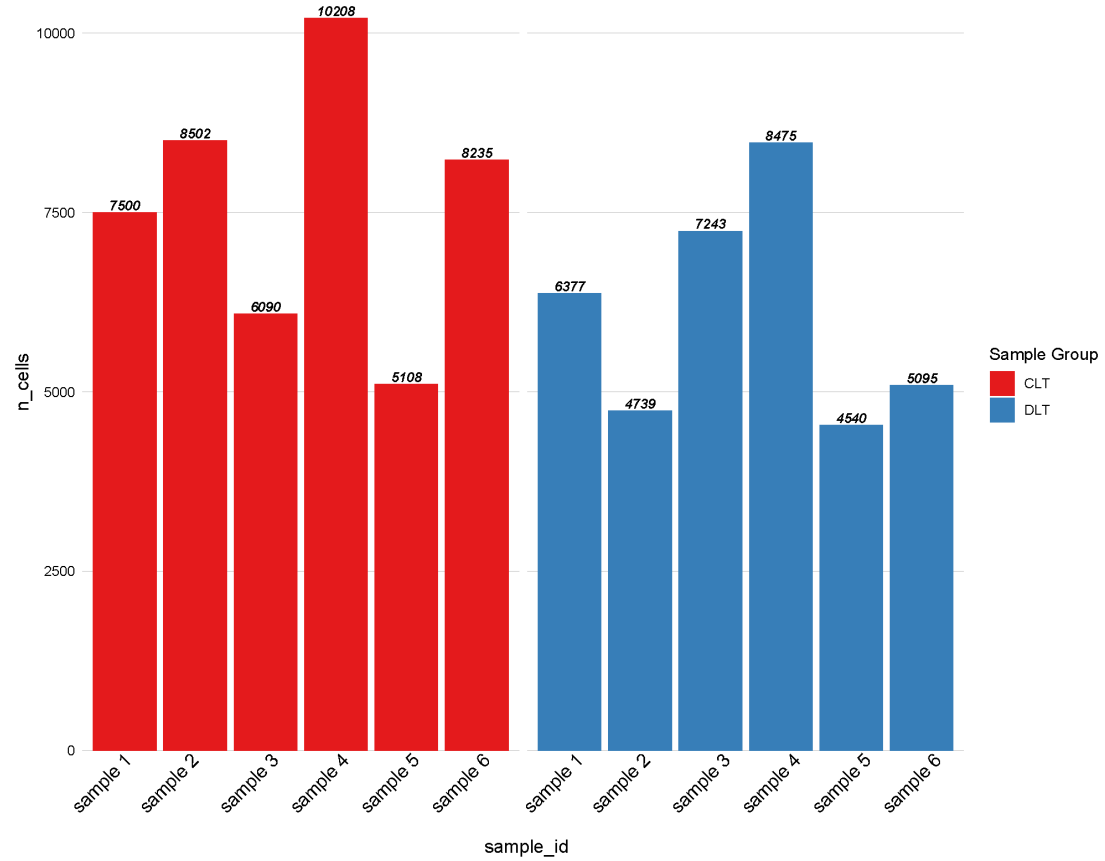

**Figure s1.** The number of cells obtained from each sample

Supplement: Supplementary file 1 [file DataSheet1.pdf]
