# Supplementary material for: Imaging mass cytometry of the immune microenvironment in alveolar echinococcosis
Source: Front Cell Infect Microbiol. 2026 May 8;16:1759455. doi: 10.3389/fcimb.2026.1759455 (PMC13194580; doi:10.3389/fcimb.2026.1759455)
Supplement: Supplementary file 2 [file DataSheet2.pdf]

Supplementary Table 1 Clinical information of 6 patients with alveolar echinococcosis for imaging mass cytometry

Samples	age	gender	infection site	lesion size（cm）	clinical staging	treatment regimen
1	13	Male	Liver Segment V (S5)	2.8×2.4	P2N0M0	newly diagnosed, untreated
2	11	Female	Liver Segments I/VI/VIII (S1/6/8)	4.0×3.2	P2N0M0	newly diagnosed, untreated
3	14	Female	Liver Segment V (S5)	3.9×2.3	P2N0M0	newly diagnosed, untreated
4	54	Female	Liver Segment V (S5)	10.4×10.2	P2N0M0	newly diagnosed, untreated
5	32	Female	Liver Segments V–VI (S5–S6)	8.0×4.9	P2N0M0	newly diagnosed, untreated
6	52	Male	Liver Segment V (S5)	12.0×10.0	P2N0M0	newly diagnosed, untreated
