# Supplementary material for: Imaging mass cytometry of the immune microenvironment in alveolar echinococcosis
Source: Front Cell Infect Microbiol. 2026 May 8;16:1759455. doi: 10.3389/fcimb.2026.1759455 (PMC13194580; doi:10.3389/fcimb.2026.1759455)
Supplement: Supplementary file 3 [file DataSheet3.pdf]

Supplementary Table 2 Antibody information

Metal	Isotope	Host Species	Antigen	Clone	Intracellular/ Surface	Vendor	Catalog#	Dilution
**141Pr**	IgG	Mouse	Alpha-SMA	1A4	**Surface**	Standard BioTools	3141017D	**1:100**
**142Nd**	IgG	Mouse	Pan-CytoKeratin	AE-1/AE-3	**Surface**	Standard BioTools	91H006142	**1:100**
**143Nd**	IgG	Rabbit	Vimentin	D21H3	**Surface**	Standard BioTools	3143027D	**1:100**
**144Nd**	IgG	Rabbit	CD14	EPR3653	**Surface**	abcam	ab226121	**1:100**
**145Nd**	IgG	Rabbit	CD45	D9M8I	**Surface**	Standard BioTools	91H009145	**1:100**
**146Nd**	IgG	Rabbit	CD163	EPR19518	**Surface**	abcam	ab213612	**1:100**
**148Nd**	IgG	Mouse	CD15	W6D3	**Surface**	biolegend	323002	**1:100**
**149Sm**	IgG	Rabbit	NKG2A	EPR23737-127	**Surface**	abcam	ab273516	**1:100**
**150Nd**	IgG	Rabbit	PD-L1	E1L3N	**Surface**	Standard BioTools	3150031D	**1:100**
**151Eu**	IgG	Rabbit	m6A	—	**Intracellular**	synaptic system	202 003	**1:100**
**152Sm**	IgG	Rabbit	CD28	EPR22076	**Surface**	abcam	ab243557	**1:100**
**153Eu**	IgG	Rabbit	TIGIT	BLR047F	**Surface**	abcam	ab243903	**1:100**
**155Gd**	IgG	Rat	FOXP3	236A/E7	**Intracellular**	Standard BioTools	3155016D	**1:100**
**156Gd**	IgG	Rabbit	CD4	EPR6855	**Surface**	Standard BioTools	3156033D	**1:100**

**158Gd**	IgG	Rabbit	E-cadherin	24E10	**Surface**	Standard BioTools	3158029D	**1:100**
**159Tb**	IgG	Mouse	CD68	KP1	**Surface**	abcam	ab233172	**1:100**
**160Gd**	IgG	Rabbit	CD1c	EPR23189-196	**Surface**	abcam	ab270797	**1:100**
**161Dy**	IgG	Rabbit	CD33	EPR23051-101	**Surface**	abcam	ab269461	**1:100**
**162Dy**	IgG	Rabbit	CD8	C8/144B	**Surface**	Standard BioTools	3162034D	**1:100**
**163Dy**	IgG	Mouse	VEGF	G153-694	**Surface**	Standard BioTools	3163028D	**1:100**
**164Dy**	IgG	Rabbit	ARG1	D4E3M	**Intracellular**	CST	89872SF	**1:100**
**165Ho**	IgG	Rabbit	PD-1	EPR4877(2)	**Surface**	Standard BioTools	3165039D	**1:100**
**166Er**	IgG	Rabbit	MPO	EPR20257	**Intracellular**	abcam	ab221847	**1:100**
**167Er**	IgG	Rabbit	GZMB	EPR20129-217	**Intracellular**	abcam	ab219803	**1:100**
**168Er**	IgG	Rabbit	CD19	EPR5906	**Surface**	abcam	ab271904	**1:100**
**170Er**	IgG	Rabbit	CD3	Polyclonal	**Surface**	Standard BioTools	3170019D	**1:100**
**172Yb**	IgG	Mouse	CD47	CD47/3019	**Surface**	abcam	ab261731	**1:100**
**173Yb**	IgG	Mouse	CD45RO	UCH-L1	**Surface**	abcam	ab23	**1:100**
**174Yb**	IgG	Mouse	HLA-DR	LN3	**Surface**	biolegend	327002	**1:100**
**175Lu**	IgG	Rabbit	CD56	EP2567Y	**Surface**	abcam	ab214435	**1:100**
