# Supplementary material for: Imaging mass cytometry of the immune microenvironment in alveolar echinococcosis
Source: Front Cell Infect Microbiol. 2026 May 8;16:1759455. doi: 10.3389/fcimb.2026.1759455 (PMC13194580; doi:10.3389/fcimb.2026.1759455)
Supplement: Supplementary file 4 [file DataSheet4.pdf]

Supplementary Table 3 Cell clustering and definition

Cluster	Cell markers	Cell
Cluster1	Pan_keratin+E_cad+	Epithelial
Cluster2	Pan_keratin+E_cad+	Epithelial
Cluster3	Pan_keratin+E_cad+	Epithelial
Cluster4	Pan_keratin(lower)+E_cad+	Epithelial
Cluster5	Pan_keratin+E_cad+	Epithelial
Cluster6	Pan_keratin+E_cad+	Epithelial
Cluster7	CD45+CD3+CD4+CD45RO-	CD4+Te
Cluster8	CD45+CD3+CD4+Foxp3+	CD4+T(Treg)
Cluster9	CD45+CD3+CD4+CD45RO-	CD4+Te
Cluster10	CD45+CD3+CD4+CD45RO-	CD4+Te
Cluster11	CD45+CD3+CD4+CD45RO-	CD4+Te
Cluster12	CD45+CD3+CD4+CD45RO-	CD4+Te
Cluster13	CD45+CD3+CD4+CD45RO-	CD4+Te
Cluster14	CD45+CD3+CD8+CD45RO-	CD8+Te
Cluster15	CD45+CD3+CD8+CD45RO-	CD8+Te
Cluster16	CD45+CD3+CD8+CD45RO-	CD8+Te
Cluster17	CD45+CD3+CD8+CD45RO-	CD8+Te
Cluster18	CD45+CD3+CD4+CD45RO+	CD4+Tem
Cluster19	CD45+CD3+CD8+CD45RO+	CD8+Tem
Cluster20	CD45+CD19+MPO+	B(B)
Cluster21	CD45+MPO+	Neutrophil
Cluster22	aSMA+Vimentin+	Stromal
Cluster23	Vimentin+	Stromal
Cluster24	Vimentin+	Stromal
Cluster25	CD45+VEGF+	Undefine(Undefine)
Cluster26	CD45+CD14+	Myeloid(Monocytes)
Cluster27	CD45+HLA_DR+	Myeloid(DC)
Cluster28	CD45+CD14+CD68+	Myeloid (Mac1)
Cluster29	CD45+CD14+CD68+CD163+	Myeloid (Mac2)
Cluster30	CD45+MPO+	Neutrophil
Cluster31	CD45+MPO+	Neutrophil
Cluster32	CD45+MPO+	Neutrophil
Cluster33	CD45+MPO+	Neutrophil
Cluster34	CD45+MPO+	Neutrophil
Cluster35	CD45+CD15+MPO+	CD15+Neutrophil
Cluster36	CD45+CD56+	NK
