# Supplementary material for: Imaging mass cytometry of the immune microenvironment in alveolar echinococcosis
Source: Front Cell Infect Microbiol. 2026 May 8;16:1759455. doi: 10.3389/fcimb.2026.1759455 (PMC13194580; doi:10.3389/fcimb.2026.1759455)
Supplement: Supplementary file 5 [file DataSheet5.pdf]

Supplementary Table 4 Clinical characteristics of patients

	**Children (10)**	**Adult(11)**
**Age**		
Years, median (minimum,maximum)	13.5(7,18)	53(32,60)
**Sex**		
Female	5(50%)	6(54%)
**WHO-IWGE PNM classification**		
P1N0M0	4	4
P2N0M0	3	4
P3N0M0	3	3
P4N0M0	0	0
